# Relationship between Systemic Vascular Characteristics and Retinal Nerve Fiber Layer Loss in Patients with Type 2 Diabetes

**DOI:** 10.1038/s41598-018-28985-8

**Published:** 2018-07-12

**Authors:** Soo Ji Jeon, Hae-Young Lopilly Park, Jae Hyung Lee, Chan Kee Park

**Affiliations:** Department of Ophthalmology, Seoul St. Mary’s Hospital, College of Medicine, The Catholic University of Korea, Seoul, Republic of Korea

## Abstract

Retinal nerve fiber layer (RNFL) loss in diabetic patients is especially common regardless of diabetic retinopathy (DR). The correlations between nonglaucomatous RNFL loss and systemic characteristics in diabetic patients have aroused interests in many aspects. 167 subjects with type 2 diabetes who underwent evaluation for arterial stiffness and cardiovascular autonomic function using heart rate variability (HRV) were included in this study. Arterial stiffness was measured using cardio-ankle vascular index (CAVI) and ankle-brachial index (ABI). Multivariate regression analysis was performed to determine factors influencing the presence of RNFL loss according to age. Factors determining the superior location of diabetic RNFL loss were also investigated. CAVI were worse in patients with RNFL loss, especially in those with old age (≥50 yrs) (*p* = 0.037). Influential factor of RNFL defect in old group was ABI (*p* = 0.007). However, in young group (<50 yrs), HRV parameter (low-frequency/high-frequency ratio) determined the presence of RNFL loss (*p* = 0.040). Significant determinants of superior RNFL defect in old subjects were CAVI and ABI (*p* = 0.032 and *p* = 0.024). For young diabetic patients, autonomic dysfunction may have relationship with RNFL loss, but as patients get older, arterial stiffness could aggravate vascular autoregulation and diabetic RNFL loss. RNFL loss in diabetes may be correlated with systemic vascular conditions.

## Introduction

Retinal nerve fiber layer (RNFL) defect refers to the loss of retinal neuronal cell^1^. It is typically observed in glaucoma patients. However, it is also seen in nonglaucomatous retina of diabetic patients. RNFL loss in diabetic patients is especially common regardless of diabetic retinopathy (DR)^2^. This suggests that RNFL loss without DR can be a type of optic neuropathy in diabetic patients^3^.

Diabetic RNFL loss is influenced by multiple factors, including oxidative stress^3^, advanced glycation end products^4^, and blocked retrograde axonal flow of retinal ganglion cells^5^. Among these factors, oxidative stress could be influenced by systemic vascular conditions. Retinal vascular circulation is controlled through autonomic nervous system^6^, and it also could be impaired by arterial stiffness through narrowing vascular caliber^7^.

Several studies have evaluated the stiffness of vessel in diabetic patients and found that systemic vascular incompetence commonly occurs, wielding an influence on diabetic complications^8,9^. Shim *et al*.^10^ have reported that increased arterial stiffness is associated with existence of open-angle glaucoma in diabetic patients using brachial-ankle pulse wave velocity (baPWV) index. In addition, increased arterial stiffness can be affected by old age itself. Increased trend of arterial stiffness is even shown in healthy subjects with increasing age^11^. So, it is natural that the older the diabetic patient is, the more affected by the vascular stiffness.

Cardiovascular autonomic function could be another factor affecting systemic circulation in diabetic patients. Indeed, cardiovascular autonomic dysfunction has been widely reported using multiple indices^12,13^. Worse heart rate variability (HRV) parameters could contribute to cardiac functional damage in diabetic patients, so we hypothesised that peripheral circulation might be influenced by compromised cardiac function, especially in retina of diabetes.

Our purpose of this study is to look into how systemic vascular changes of diabetes can affect retina, and to investigate the correlations between nonglaucomatous RNFL loss and systemic vascular characteristics in patients with type 2 diabetes according to aging.

## Results

Of a total of 167 subjects, 105 had RNFL losses while 62 had no RNFL loss as age- and sex- matched controls. Baseline characteristics of both groups are summarized in Table 1. There were no statistically significant differences in mean age, ratio of gender, presence of hypertension, diabetic duration, percentage of diabetic retinopathy, laboratory findings, arterial stiffness indices, and HRV parameters.

**Table 1:** Baseline characteristics of T2DM patients without or with RNFL loss.

	Without RNFL Loss (n = 62)	With RNFL Loss (n = 105)	*P* Value
Age, years	57.55 (±13.55)	55.26 (±13.15)	0.284*
Sex, male:female	34:27	46:60	0.102^‡^
Hypertension, n (%)	25 (40.3%)	48 (45.3%)	0.531^‡^
DM duration, years	11.65 (±7.20)	12.77 (±7.34)	0.335*
Diabetic retinopathy, n (%)	24 (38.7%)	53 (48.6%)	0.156^‡^
Laboratory findings			
HbA1c	7.91 (±1.88)	8.38 (±2.25)	0.157*
eGFR	92.15 (±30.98)	89.74 (±25.90)	0.593*
Total cholesterol	162.52 (±44.42	168.12 (±45.65)	0.443*
Triglyceride	158.06 (±205.70)	134.26 (±96.76)	0.395*
HDL	47.53 (±15.67)	46.86 (±12.53)	0.764*
LDL	85.96 (±31.12)	95.39 (±37.48)	0.114*
CAVI	8.07 (±1.23)	8.40 (±1.58)	0.157*
ABI	1.05 (±0.09)	1.02 (±0.13)	0.254*
Presence of carotid plaque, n (%)	14 (26.9%)	35 (33.1%)	0.239^‡^
SDNN	69.57 (±295.42)	29.47 (±22.88)	0.338*
LF/HF	3.66 (±4.06)	3.20 (±5.46)	0.605*

^*^Student t-test.

^‡^Chi-square test.

HbA1c: glycosylated hemoglobin; eGFR: estimated glomerular filtration rate; HDL: high-density lipoprotein; LDL: low-density lipoprotein; CAVI: cardio-ankle vascular index; ABI: ankle-brachial index.

Data are mean (±SD) or number (%), as appropriate.

### Subdividing subjects according to age

In consideration of the effect of aging on vessel wall, arterial stiffness indices were compared after subdividing patients into old and young groups. According to American Diabetes Association (ADA) 2016 guidelines^14^, there was different criteria of considering antiplatelet agents to prevent atherosclerotic cardiovascular disease (ASCVD) based on age 50. So we determined the cutoff point of grouping subjects as age of 50. In young age group, there were no significant differences in mean value of CAVI, ABI, and ratio of patients with abnormal arterial stiffness index (Table 2).

**Table 2:** Comparison of arterial stiffness index according to age.

	Without RNFL Loss (n = 62)	With RNFL Loss (n = 105)	*P* Value
Old (≥50 yrs)			
N	48	69	
CAVI	8.38 (±1.05)	8.92 (±1.53)	0.037*
ABI	1.06 (±0.10)	1.01 (±0.14)	0.051*
Abnormal CAVI, n (%)	16 (33.3%)	35 (50.7%)	0.062^‡^
Abnormal ABI, n(%)	3 (6.6%)	13 (18.8%)	0.051^‡^
Presence of carotid plaque, n (%)	13 (34.2%)	30 (48.4%)	0.165^‡^
Young (<50 yrs)			
N	14	36	
CAVI	6.98 (±1.22)	7.43 (±1.19)	0.237*
ABI	1.00 (±0.08)	1.04 (±0.10)	0.226*
Abnormal CAVI, n (%)	1 (7.1%)	3 (8.3%)	0.889^‡^
Abnormal ABI, n(%)	2 (14.3%)	2 (5.6%)	0.307^‡^
Presence of carotid plaque, n (%)	1 (7.1%)	5 (14.7%)	0.471^‡^

^*^Student t-test.

^‡^Chi-square test.

CAVI: cardio-ankle vascular index; ABI = ankle-brachial index.

On the other hand, in the old age group, there were statistically significant or considerable differences. In old patients who had RNFL loss, mean arterial stiffness indices were worse (CAVI: 8.38 vs. 8.92; ABI: 1.06 vs. 1.01) and ratios of subjects who had abnormal CAVI or ABI results were also higher compared to subjects without RNFL loss (CAVI: 50.7% vs. 33.3%; ABI: 18.8% vs. 6.6%, Table 2). The *P* value was significant in CAVI value, but mean ABI and ratio of subjects with abnormal ABI showed marginal *P* values (0.051 and 0.051).

Multivariate regression analysis including HRV parameters and arterial stiffness indexs was performed for subgroup analysis. Table 3 shows the results of statistically significant multivariate logistic regression analysis model of each group. In the old age group, only ABI had significant effect on RNFL loss (β = −11.057, *p* = 0.007). Values of autonomic functional evaluation, such as SDNN (standard deviation of the NN intervals) and LF/HF (low-frequency/high-frequency), did not show any significant or meaningful effect. LF/HF ratio were statistically significant in multivariate model analysis in the young age group (β = −0.170, *p* = 0.040). In this group, HbA1c level was also meaningful for diabetic RNFL loss (β = 0.391, *p* = 0.033).

**Table 3:** Multivariate regression analysis of RNFL loss in old and young age groups including autonomic nerve function indices (SDNN and LF/HF), arterial stiffness indices (CAVI and ABI) and laboratory findings.

	Old (≥50 yrs)				Young (<50 yrs)	
	β	95% CI	*P* Value	β	95% CI	*P* Value
Duration of DM	−0.010	0.891 to 1.100	0.857	0.128	0.990 to 1.305	0.069
SDNN	0.052	0.997 to 1.114	0.062	−0.026	0.931 to 1.019	0.255
LF/HF	0.033	0.818 to 1.306	0.783	−0.170	0.717 to 0.992	0.040
HbA1c	0.075	0.592 to 1.963	0.806	0.391	1.031 to 2.119	0.033
eGFR	0.007	0.969 to 1.047	0.723	−0.001	0.969 to 1.030	0.967
Total cholesterol	−0.134	0.720 to 1.064	0.180	0.039	0.959 to 1.127	0.345
TG	0.032	0.990 to 1.078	0.139	−0.009	0.977 to 1.005	0.195
HDL	0.227	0.981 to 1.605	0.071	−0.090	0.832 to 1.003	0.059
LDL	0.147	0.952 to 1.409	0.142	−0.035	0.886 to 1.052	0.423
CAVI	0.222	0.550 to 2.835	0.596	−0.061	0.541 to 1.636	0.828
ABI	−11.057	0.000 to 0.046	0.007	4.258	0.191 to 26166.7	0.158

Enter mode was used for this logistic regression analysis.

CI: confidence interval.

SDNN: standard deviation of normal to normal intervals in heart rate variability; LF/HF: ratio of low-frequency power to high-frequency power in heart rate variability; HbA1c: glycosylated hemoglobin; eGFR: estimated glomerular filtration rate; TG: triglyceride; HDL: high-density lipoprotein; LDL: low-density lipoprotein; CAVI: cardio-ankle vascular index; ABI: ankle-brachial index.

### Discrimination of RNFL location

Among patients with RNFL loss, the location of RNFL defect was also classified. Results on their correlations with arterial stiffness indices are summarized in Table 4. In the young age group, no significant difference in arterial stiffness indices was found between superior and inferior RNFL loss subjects. In the old age group, the ratio of patients with abnormal CAVI values was higher (60.5 vs. 34.6%) in subjects with superior RNFL loss compared to that in subjects with inferior RNFL loss. Mean CAVI was worse (9.20 vs. 8.46) in superior RNFL loss subjects, but the *p* value was marginally insignificant (p = 0.054). ABI value or ratio of abnormal ABI patients was not significantly different between superior and inferior RNFL loss groups.

**Table 4:** Comparison of arterial stiffness index among patients with RNFL loss according to RNFL loss location (superior or inferior).

	Superior RNFL Loss	Inferior RNFL Loss	*P* Value
Old (≥50 yrs)			
N	43	26	
CAVI	9.20 (±1.59)	8.46 (±1.32)	0.054*
ABI	1.03 (±0.12)	0.98 (±0.17)	0.212*
Abnormal CAVI, n (%)	26 (60.5%)	9 (34.6%)	0.037^‡^
Abnormal ABI, n(%)	7 (16.3%)	6 (23.1%)	0.484^‡^
Presence of carotid plaque, n (%)	17 (44.7%)	13 (54.2%)	0.469^‡^
Young (<50 yrs)			
N	29	7	
CAVI	7.45 (±1.23)	7.35 (±1.09)	0.843*
ABI	1.04 (±0.11)	1.04 (±0.08)	0.981*
Abnormal CAVI, n (%)	3 (10.3%)	0 (0.0%)	0.374^‡^
Abnormal ABI, n(%)	2 (6.9%)	0 (0.0%)	0.475^‡^
Presence of carotid plaque, n (%)	4 (14.3%)	1 (16.7%)	0.881^‡^

^*^Student t-test.

^‡^Chi-square test.

CAVI: cardio-ankle vascular index; ABI: ankle-brachial index.

As shown in Table 5, logistic regression analysis was done to discriminate meaningful factor affecting the existence of superior RNFL loss in old group. The CAVI and ABI were associated significantly (p = 0.032 and 0.024, respectively) in multivariate logistic regression analysis, but autonomic functional indices such as SDNN and LF/HF did not show significant effect on superior RNFL loss in old group.

**Table 5:** Univariate and multivariate regression analysis of superior RNFL loss in old age group (≥50 yrs).

	Univariate Analysis			Multivariate Analysis*		
	β	95% CI	*P* Value	β	95% CI	*P* Value
Duration of DM	0.026	0.962 to 1.094	0.435			
SDNN	0.004	0.984 to 1.024	0.709			
LF/HF	0.016	0.939 to 1.101	0.686			
HbA1c	0.131	0.913 to 1.424	0.247	0.122	0.802 to 1.592	0.486
eGFR	−0.014	0.954 to 1.020	0.431			
Total cholesterol	−0.003	0.985 to 1.009	0.619			
TG	0.004	0.995 to 1.013	0.418			
HDL	−0.019	0.944 to 1.020	0.330			
LDL	−0.002	0.985 to 1.012	0.809			
CAVI	−0.356	0.483 to 1.015	0.060	−0.492	0.390 to 0.959	0.032
ABI	−2.163	0.004 to 3.500	0.215	−4.668	0.000 to 0.545	0.024

^*^Variables with *P* value less than 0.3 in univariate analysis were included.

CI: confidence interval.

SDNN: standard deviation of normal to normal intervals in heart rate variability; LF/HF: ratio of low-frequency power to high-frequency power in heart rate variability; HbA1c: glycosylated hemoglobin; eGFR: estimated glomerular filtration rate; TG: triglyceride; HDL: high-density lipoprotein; LDL: low-density lipoprotein; CAVI: cardio-ankle vascular index; ABI: ankle-brachial index.

## Discussion

Diabetic retinopathy and nephropathy can be caused by microvascular impairment due to oxidative stress with advanced glycation end products (AGEs)^15^. In the kidney, oxidative stress can cause endothelial injury, podocyte loss, albuminuria, and glomerular failure in sequence^16^. Similar microvascular endothelial structural impairment also occurs in retinal vessels. Therefore, this structural problem of vessel is a crucial factor that determines autoregulation of retinal circulation.

Autonomic neuropathy in diabetes is also important complication. It can affect cardiovascular function and increase the risk of morbidity and mortality. Dysfunction of axon and dendrite of sympathetic ganglion has been identified by multiple experiments^17^. Activation of the AGE, protein kinase C (PKC), polyol, poly(ADP-ribose) polymerase, and hexosamine pathways can contribute to metabolic imbalance in hyperglycemic environment^18^. Because arteriovenous anastomoses of capillaries are usually under sympathetic control, compromised autonomic function in diabetic patients could affect microvascular circulation.

We have been focusing on the suggestion that retinal vascular homeostasis in diabetic patient is maintained through both autoregulation and autonomic regulation of vessel. To evaluate vascular autoregulation that is associated with structural changes of vessel, arterial stiffness indices such as CAVI and ABI were selected in this study. HRV parameters were also included to assess the autonomic functional status of vessels in diabetic subjects.

CAVI has been recently proposed as an indicator of arterial stiffness and arteriosclerosis independent of arterial blood pressure^19^. Kim *et al*. have suggested that CAVI was related to smooth muscle contraction status of vessel^20^. In addition, ABI has been widely used to estimate vascular status as a simple and useful method^21^. ABI was known as a good index of atherosclerosis and lower ABI has been reported to be useful in the diagnosis of peripheral arterial disease^22,23^. Also, higher ABI was reported to represent medial artery calcification and to be related with the risk of cardiovascular event^24,25^.

Autoregulation of retinal vessel can be attained by modulating vascular tone which could be achieved through multiple mechanisms affecting vascular structures including smooth muscle cells^26^. Changes of vascular structures in old age could affect retinal vascular autoregulation. Indeed, it has been reported that smooth muscle cells of arterioles have fibrosis, degradation and decreased cross-sectional area due to aging^27,28^.

Several studies have reported that diabetic RNFL loss is more frequently found at the superior hemisphere than that at the inferior hemisphere^3,29,30^. Compromised vascular responsiveness upon metabolic imbalance^31^ in diabetic patients could explain why microaneurysm, acellular capillary, and nerve fiber loss are common in superior retinal region. Under diabetic condition, impaired vascular response could bring about deficient homeostasis of blood circulation at superior retina which may have decreased blood volume due to gravitational effect^3,32^. In our study, when RNFL loss location was classified as superior and inferior in old age group, the subjects with superior RNFL loss presented worse CAVI values (Table 4), and, statistically significant determinants of superior RNFL loss were CAVI and ABI values (Table 5). Those are at the same line of thought that the impaired autoregulation in retinal arteriole impairs retinal circulation, and it exert influence on retinal neuronal loss more at superior hemisphere.

However, in spite of relatively better vascular tone, RNFL loss can still occurs in younger diabetic patients. Our results presented that cardiovascular autonomic functional indices were only associated with RNFL loss in young subjects (Table 3). It has been reported that low SDNN of diabetic patient was associated with subclinical cardiovascular problem such as left ventricular hypertrophy^33^. Canani *et al*. suggested that diabetic patients with low HRV parameters including LF/HF ratio had more peripheral arterial disease and it could reflect dysfunction of vascular modulation through autonomic system. As above, diabetic patients are known to have increased risk of cardiovascular autonomic dysfunction in many aspects and autonomic dysfunction of vessels could explain the RNFL loss in young patients with relatively good vascular autoregulation. When meaningful factors of diabetic RNFL loss were compared between subgroups according to age, this explanation is still applicable.

Systemic vascular conditions and associated cardiovascular disease are strong predictors of mortality and prognosis in diabetic patients^34,35^. Our results implied that diabetic RNFL loss could be used as a surrogate of systemic vascular characteristics. RNFL loss in diabetic retina might represent blood flow impairment and precise optic disc evaluation in each diabetic patient may be helpful for improving the prognosis of this chronic and multisystemic disease.

## Materials and Methods

### Study design and Population

This retrospective and cross-sectional study was performed according to the tenets of the Declaration of Helsinki. It was approved by Institutional Review and Ethics Boards of Seoul St. Mary’s Hospital, South Korea. The need for written informed consent was waived by our Review Board.

A total of 167 subjects with type 2 diabetes who underwent evaluation for arterial stiffness and cardiovascular autonomic function from January 2008 to March 2015 at Seoul St. Mary’s Hospital were included in this study. If both eyes had diabetic RNFL losses, the right eye was selected. All eyes had no signs of glaucomatous optic disc morphology such as narrowing or disappearance of neuroretinal rim, disc hemorrhage, or cup-to-disc ratio asymmetry >0.2. All subjects included in this study had open angle on gonioscopy and intraocular pressure of lower than 21 mmHg without history of periorbital trauma. Excluded patients were one of following cases: type 1 diabetes, active ocular disease such as uveitis, history of retinal disease, or history of ocular surgery other than simple cataract extraction.

All subjects underwent complete ophthalmic examination including visual acuity, Goldmann applanation tonometry, slit-lamp examination, gonioscopy, red-free RNFL photography (CF-60UD; Canon, Tokyo, Japan). Best corrected visual acuity was 20/40 or better and spherical equivalent was within ± 5.0 diopters in all subjects.

Eyes without diabetic retinopathy (DR) or mild nonproliferative diabetic retinopathy (NPDR) were included. Stage of DR was evaluated by a retinal specialist (J.H.L) through fundus photography based on Early Treatment Diabetic Retinopathy Study (ETDRS) classification. Eyes with clinically significant macular edema were also excluded^36^.

**Figure 1:**
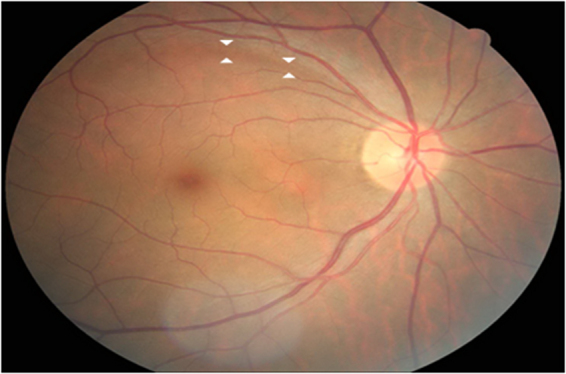
Representative case of diabetic retinal nerve fiber layer (RNFL) loss A 46-year-old male with type 2 diabetes had RNFL defect in superior portion of retina. The morphology of optic disc was not glaucomatous. White arrows demarcate the border of RNFL loss.

Only well centered retinal photographs were used to evaluate the existence of RNFL loss. These photographs were obtained using digital fundus camera at 60° view (Fig. 1). Two glaucoma specialists (S.J.J. and H.Y.P.) evaluated the fundus photograph of each patient in a blind manner. If there was disagreement between the two observers, the eye was excluded from our study. Localized diabetic RNFL loss was defined as followings: (1) width larger than a major retinal vessel at a distance of 1-disc diameter from the edge of the disc, (2) diverged in an arcuate or wedge shape, (3) reaching the edge of the disc with clear margin. Photographs containing multiple RNFL defects were excluded in order to compare difference in vascular index according to the location of RNFL loss: superior RNFL loss and inferior RNFL loss.

### Systemic examinations

Patients were diagnosed as diabetes if fasting plasma glucose level was more than 126 mg/dl, or symptoms of diabetes appeared with random plasma glucose level of more than 200 mg/dl, or HbA1c was more than 6.5%^37^. Hypertension was defined when systolic blood pressure was ≥140 mmHg and diastolic blood pressure was ≥90 mmHg, or any antihypertensive medication was used^38^.

After patients had fasted for 12 hours, blood samples were collected and blood glucose was measured using an automated enzymatic method. Glycated hemoglobin (HbA1c) level was measured to evaluate the controlled state of diabetes.

Lipid profiles (total cholesterol, triglycerides, low density lipoprotein, and high density lipoprotein) were measured and glomerular filtration rate was estimated using the Modification of Diet in Renal Disease Study equation^39^.

### Measurement of arterial stiffness indices

The arterial stiffness was measured using cardio-ankle vascular index (CAVI) and ankle-brachial index (ABI). CAVI and ABI values were obtained with a VaSera VS-1000 device (Fukuda Denshi, Tokyo, Japan).

CAVI was calculated using the distance from the level of aortic valve to the measured area and delayed time from closing of aortic valve to arterial pressure change at measure point^40^. ABI was calculated at each leg, dividing the higher pressure of posterior tibial or dorsalis pedis artery by systolic pressure at brachial artery^41^. The presence of plaque in carotid artery was distinguished using carotid sonography (HP 5500, Hewlett-Packard, USA).

### Evaluation of cardiovascular autonomic function

Cardiovascular autonomic nervous function was evaluated with parameters of heart rate variability, including standard deviation of normal to normal intervals (SDNN) and ratio of low-frequency power to high-frequency power (LF/HF). HRV evaluation was done as follows^42^. Echocardiography signals of all patients were documented after 30 minutes of rest. They were then transferred to Medicore Heart Rate Analyzer, Model SA-3000P (Medicore, Seoul, Korea).

Time domain methodologic evaluation was done through SDNN, the standard deviation of the normal rate-to-rate intervals mainly reflecting parasympathetic status. Frequency domain methodologic evaluation included LF/HF ratio. LF reflects both parasympathetic and sympathetic function while HF reflects parasympathetic dysfunction using vagal nervous compartment. Therefore, the ratio of LF/HF primarily reflects the imbalance of autonomic nervous system^43,44^.

### Statistical analysis

All statistical analyses were performed with SPSS version 19.0 (SPSS Inc., Chicago, IL). Student t tests and Chi-square tests were used to evaluate clinical characteristics between RNFL loss group and the group without showing RNFL loss. Comparison was also done dividing subjects into age-specific groups. Patients with RNFL loss were also grouped according to the location of RNFL defect (superior and inferior). Univariate and multivariate logistic regression analyses were performed to determine significant factors influencing diabetic RNFL defect. In all analysis, *P* < 0.05 was considered statistically significant.
